# Upregulation and Identification of Antibiotic Activity of a Marine-Derived *Streptomyces* sp. via Co-Cultures with Human Pathogens

**DOI:** 10.3390/md15080250

**Published:** 2017-08-11

**Authors:** Anne A. Sung, Samantha M. Gromek, Marcy J. Balunas

**Affiliations:** Division of Medicinal Chemistry, Department of Pharmaceutical Sciences, University of Connecticut, Storrs, CT 06269, USA; anne.sung@uconn.edu (A.A.S.); samantha.gromek@uconn.edu (S.M.G.)

**Keywords:** mixed fermentation, co-culture with human pathogens, microbial natural product drug discovery, antibiotic secondary metabolites

## Abstract

Marine natural product drug discovery has begun to play an important role in the treatment of disease, with several recently approved drugs. In addition, numerous microbial natural products have been discovered from members of the order Actinomycetales, particularly in the genus *Streptomyces*, due to their metabolic diversity for production of biologically active secondary metabolites. However, many secondary metabolites cannot be produced under laboratory conditions because growth conditions in flask culture differ from conditions in the natural environment. Various experimental conditions (e.g., mixed fermentation) have been attempted to increase yields of previously described metabolites, cause production of previously undetected metabolites, and increase antibiotic activity. Adult ascidians—also known as tunicates—are sessile marine invertebrates, making them vulnerable to predation and therefore are hypothesized to use host-associated bacteria that produce biologically active secondary metabolites for chemical defense. A marine-derived *Streptomyces* sp. strain PTY087I2 was isolated from a Panamanian tunicate and subsequently co-cultured with human pathogens including *Bacillus subtilis*, methicillin-sensitive *Staphylococcus aureus* (MSSA), methicillin-resistant *Staphylococcus aureus* (MRSA), and *Pseudomonas aeruginosa*, followed by extraction. Co-culture of *Streptomyces* sp. PTY087I2 with each of these human pathogens resulted in increased production of three antibiotics: granaticin, granatomycin D, and dihydrogranaticin B, as well as several analogues seen via molecular networking. In addition, co-cultures resulted in strongly enhanced biological activity against the Gram positive human pathogens used in these experiments. Expanded utilization of co-culture experiments to allow for competitive interactions may enhance metabolite production and further our understanding of these microbial interactions.

## 1. Introduction

Early drug discovery from natural products focused primarily on plants, but after the discovery and development of penicillin, scientists started looking at natural products from microorganisms [1]. Natural products produced by microbes have played a significant role in drug discovery and the treatment of diseases [2], with many of the small molecule drugs launched from 1981 to 2006 derived from microbial natural products [3]. However, although there is a large cadre of antimicrobial drugs available, new antimicrobial drugs are still urgently needed due to increasing drug resistance in pathogenic bacteria [4,5,6,7].

Host-associated microbes have been the focus of many natural product drug discovery efforts, most notably involving the investigation of bacteria associated with marine sponges [8,9,10], as well as more recent efforts studying microbial symbionts associated with other marine invertebrates such as corals, molluscs, bryozoans, crustaceans, and tunicates. Tunicates—also known as urocordates, ascidians, or sea squirts—are a marine invertebrate member of the Chordata phylum and one of our closest living invertebrate relatives [11,12]. Tunicates are known to harbor photosymbiotic cyanobacteria, generally found within their tunic or carried by larvae [13,14,15], and more recent studies have indicated that the tunicate microbial community is a complex array of cyanobacteria, heterotrophic bacteria, and fungi [16,17,18], capable of producing diverse and biologically-active secondary metabolites.

The myriad interactions that occur between microbes, their invertebrate hosts and the surrounding ecosystem, have been shown to be challenging to replicate in laboratory cultures [19,20,21,22]. Microorganisms interact with each other, their hosts, and their environment, and these interactions are thought to be one of the driving forces of the production of bioactive secondary metabolites [22], e.g., in response to pathogenic fungi or bacteria, or due to environmental conditions such as limited nutrients or space. Genomic analyses have revealed an abundance of biosynthetic gene clusters capable of producing secondary metabolites, but only a small portion are commonly expressed under laboratory monoculture conditions. Mixed fermentation is one of the techniques that has been explored to maximize bacterial chemical diversity and has been shown to increase yields of previously described metabolites, cause production of previously undetected metabolites, and/or increase antibiotic activity of co-cultured extracts [3,23]. The competitive interactions in these mixed fermentation co-cultures may induce previously silent biosynthetic pathways, enhancing production of secondary metabolites in laboratory conditions.

During the course of our investigation of new antimicrobial natural products from tunicate-associated bacteria, we collected a Panamanian tunicate *Styela canopus* from which we isolated a *Streptomyces* sp., designated as strain PTY087I2, that exhibited potent antimicrobial activity against a suite of bacterial pathogens. Actinobacteria, especially the *Streptomyces* species, have been abundant sources of chemically diverse and biologically active natural products, and have been shown to be well adapted to living in association with marine invertebrates, including tunicates [24,25,26]. While studying the biologically active metabolites from *Streptomyces* sp. PTY087I2, we also completed a whole genome sequence and discovered extensive secondary metabolite biosynthetic potential [27], much of which was not evident in our initial metabolomic analyses. We therefore initiated mixed fermentation co-culture experiments with four human bacterial pathogens [three Gram-positive strains including *Bacillus subtilis* (BS), methicillin-sensitive *Staphylococcus aureus* (MSSA), and methicillin-resistant *Staphylococcus aureus* (MRSA), as well as one Gram-negative strain *Pseudomonas aeruginosa* (PA)] in order to test the hypothesis that these human pathogens may cause *Streptomyces* sp. PTY087I2 to increase production of antibacterial secondary metabolites, specifically inducing production of compounds that may then increase biological activity against these same pathogens. Herein, we report the genomic capacity for secondary metabolite production of a new bacterial strain, *Streptomyces* sp. PTY087I2, as well as co-culture experiments designed to induce production of antibacterial compounds from this strain.

## 2. Results and Discussion

### 2.1. Isolation and Identification of *Streptomyces* sp. PTY087I2

A solitary brown tunicate with striations on the siphons was collected from mangrove roots in the Bastimentos National Park in Bocas del Toro, Panama (Figure S1). The dissected tunicate was identified via morphological comparison as *Styela canopus* (Rosana Rocha, personal communication). After ethanol surface sterilization, the tunicate was rinsed with sterile seawater and plated onto R2A media for bacterial isolation, upon which the actinomycete *Streptomyces* sp. PTY087I2 was isolated. On nutrient rich YSP agar (yeast, starch, peptone, described below), PTY087I2 exhibited white aerial mycelia—typical of actinomycetes—as well as secreting deep purple pigment into the agar (Figure S1).

A full genome sequence was obtained for this strain (GenBank accession number LZRD00000000) and the strain was determined to be in the *Streptomyces* genus [27]. The closest genome was determined to be *Streptomyces roseosporus* NRRL 11379 (accession number ABYX00000000), which had 94.7% bi-directional average nucleotide identity (ANI) compared with *Streptomyces* sp. PTY087I2 [27]. This level of genomic divergence suggests that strain PTY087I2 should be classified as a new species of *Streptomyces*.

### 2.2. Secondary Metabolite Biosynthetic Potential of *Streptomyces* sp. PTY087I2

The *Streptomyces* sp. PTY087I2 genome was subjected to analysis using the antibiotics and Secondary Metabolite Analysis Shell (antiSMASH [28]) to determine the secondary metabolite biosynthetic capacity of this strain. Overall, antiSMASH analysis indicated that PTY087I2 has strong potential to produce a wide variety of secondary metabolites with 37 biosynthetic gene clusters (BGCs) identified from a broad range of structural classes including non-ribosomal peptide synthetase (NRPS), polyketide synthase (PKS) including Type I, II, and III, terpenoid, bacteriocidin, ectoine, siderophore, melanin, lantipeptide, lassopeptide, thiopeptide, butyrolactone, arylpolyene, and several types of hybrid compounds (e.g., NRPS-PKS) (Table 1). The majority of these BGCs were similar to 30 of 37 known clusters, with the remaining seven having 100% similarity including for SGR PTMs, melanin, ectoine, griseobactin, SRO15-2005 (lassopeptide), SRO15-2005 (NRPS), and amfS biosynthesis (Table 1, molecular weights of the first compound in each of the identified BGC series are provided for comparison with metabolomic profiles of this strain).

### 2.3. Secondary Metabolite Production by *Streptomyces* sp. PTY087I2 Mono- and Co-Cultures

*Streptomyces* sp. PTY087I2 was cultured using a small-scale culturing process to afford a dark blue monoculture that was extracted to afford an organic extract that was analyzed by Liquid Chromatography–Mass Spectrometry (LC–MS) (Figure S2). Unexpectedly, extracts of PTY087I2 monocultures did not exhibit nearly the level of secondary metabolite production that was expected based on the biosynthetic potential that was evident in the genome. Thus, co-cultures were initiated with four human pathogens, *Bacillus subtilis* (BS), methicillin-sensitive *Staphylococcus aureus* (MSSA), methicillin-resistant *Staphylococcus aureus* (MRSA), and *Pseudomonas aeruginosa* (PA), in an attempt to induce biologically relevant secondary metabolite production. 

**Figure 1:**
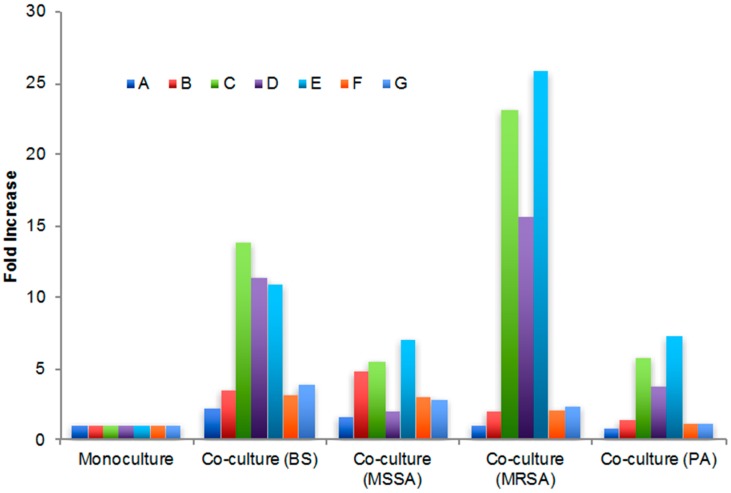
Fold increase of peak area for peaks A–G from *Streptomyces* sp. PTY087I2 monoculture and co-culture with human pathogens (BS = *Bacillus subtilis*; MSSA = methicillin-sensitive *Staphylococcus aureus*; MRSA = methicillin-resistant *Staphylococcus aureus*; PA = *Pseudomonas aeruginosa*).

Extracts of the co-cultures were analyzed by LC–MS and the UV chromatogram of each co-culture extract was overlaid with the UV chromatogram of the monoculture (Figure S2A–D). Based on increased production in co-cultures, the peaks of interest were determined to elute at 5, 10, 11, 13, 16, 34, and 68 min and labeled A–G (Figure S2A–D). The areas under the peaks were quantified (Table 2) and the fold increase for these peaks was calculated (Figure 1). The most substantial fold increases were seen for peaks C–E, particularly for the PTY+MRSA co-cultures, with 23.1, 15.6, and 25.9-fold increases in area, respectively. Peaks C and E were significantly higher in all co-culture extracts as compared with monoculture extracts (Figure 1), while peak D was substantially higher for all but the PTY+MSSA co-culture where production was only slightly increased. Production of peaks F and G was weakly increased for co-cultures with the Gram-positive pathogens (BS, MSSA, and MRSA), while peak A was only increased when co-cultured with BS and MSSA. Interestingly, co-culture with the Gram-negative pathogen PA typically resulted in less upregulation of secondary metabolite production than with the Gram-positive organisms.

**Figure 2:**
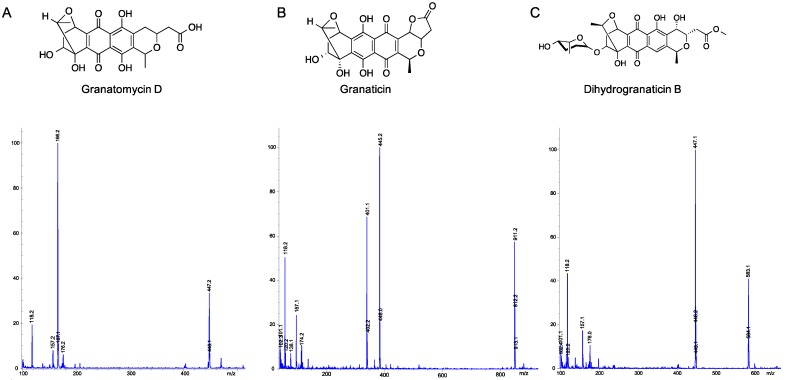
Mass spectral identification of peaks C–E as granatomycin D, granaticin, and dihydrogranaticin B, respectively. (**A**) Peak C eluted at retention time (t_R_) 11.4 min, with [M + H]^+^ of 447.2, consistent with a molecular formula of C_22_H_23_O_10_, confirming production of granatomycin D (446.4 g/mol); (**B**) Peak D eluted at t_R_ 13.2 min, with [M + H]^+^ of 445.2, consistent with a molecular formula of C_22_H_21_O_10_, confirming production of granaticin (444.4 g/mol); (**C**) Peak E eluted at t_R_ 17.0 min, with [M + Na]^+^ of 583.1, consistent with a molecular formula of C_28_H_32_O_12_Na, confirming production of dihydrogranaticin B (560.5 g/mol).

After observing substantial upregulation of several peaks in the co-cultured samples, mass spectrometry was used for identification of peaks C–E (Figure 2). Peak C eluted at 11.4 min with an [M + H]^+^ 447.2, peak D eluted at 13.2 min with an [M + H]^+^ 445.2, and peak E eluted at 17.0 min with an [M + Na]^+^ 583.1, consistent with the molecular weights and fragmentation patterns of granatomycin D (446.40 g/mol), granaticin (444.39 g/mol), and dihydrogranaticin B (560.55 g/mol), respectively (Figure 2), confirmed by high resolution mass spectrometry (Figure S3). Granaticin, a pyranonaphthoquinone originally isolated from *Streptomyces olivaceus*, is known to be produced by other actinomycetes [29], and demonstrates strong antibacterial activity against Gram-positive bacteria but has little or no activity against Gram-negative bacteria. Granatomycin D and dihydrogranaticin B lack the furanone ring of granaticin, which is replaced by a methyl ester, with dihydrogranaticin B also having a glycosylation. Like granaticin, granatomycin D has been shown to have antibiotic activity against Gram-positive bacteria [30] while no activity has previously been reported for dihydrogranaticin B.

**Figure 3:**
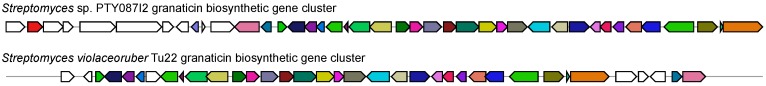
Comparison of granaticin biosynthetic gene clusters from *Streptomyces* sp. PTY087I2 and *S. violaceoruber* TÜ22. The granaticin biosynthetic gene cluster from *Streptomyces* sp. PTY087I2 was compared to that from *S. violaceoruber* TÜ22 and showed 83% similarity. Figure adapted from antiSMASH output [28].

Given the chemical identification of granaticin and derivatives, we reexamined the genome sequence and found a Type II PKS with 83% similarity to the granaticin BGC from *Streptomyces violaceoruber* TÜ22 (Figure 3) [31]. The granaticin BGC in *Streptomyces* sp. strain PTY087I2 contains 22 of the 37 biosynthesis genes with similar genome arrangement as previously described for *Streptomyces violaceoruber* TÜ22 (Table S1) [31].

### 2.4. Antibacterial Activity against Challenge Pathogens

Monoculture and co-culture extracts were all tested for antibacterial activity against the original challenge pathogens (Table 3) and while none of the extracts exhibited activity against *Pseudomonas aeruginosa* [minimum inhibitory concentration (MIC) >400 μg/mL], all showed equal or increased activity against the Gram-positive pathogens. The co-culture with MRSA resulted in the most significant increases in biological activity with MIC values 16X, 4X, and 8X more potent than the monoculture against *Bacillus subtilis*, MSSA, and MRSA, respectively (Table 3). The increased biological activity of the PTY+MRSA extract is consistent with the significantly increased metabolite production seen for the three naphthoquinone antibiotics (Figure 1). In addition to the increased activity seen for the MRSA co-culture, increases were also seen with co-culture with BS (4X, 2X, and 2X more potent against BS, MSSA, and MRSA, respectively), although co-culture with MSSA did not result in increased biological activity, possibly related to the lower overall increases in secondary metabolite production, especially for peak D, granaticin. Interestingly, co-culture with the Gram-negative bacterium *Pseudomonas aeruginosa* (PA) resulted in a 4-fold increase in potency against all Gram-positive pathogens but still did not result in activity against *Pseudomonas aeruginosa* (Table 3).

### 2.5. Utilization of Molecular Networks to Identify Other Naphthoquinone Derivatives

**Figure 4:**
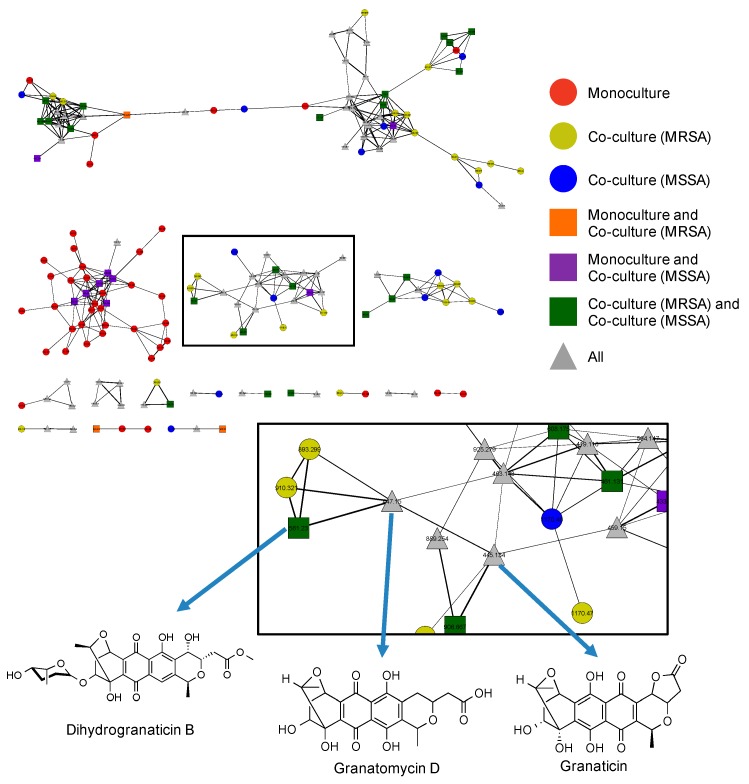
Utilization of molecular networks to identify naphthoquinone derivatives from extracts of *Streptomyces* sp. PTY087I2 monoculture (red), PTY087I2 co-cultured with MRSA (yellow), and PTY087I2 co-cultured with MSSA (blue). Highlighted area includes nodes for granatomycin D, granaticin, and dihydrogranaticin B, as well as indicating presence of several derivatives of these compounds in one or more extracts.

To further explore production of other related naphthoquinone derivatives that might be involved in the observed antibacterial activity we performed MS/MS molecular networking [32]. Our molecular network consisted of MS/MS data from three bacterial extracts, including the PTY monoculture, the PTY+MRSA co-culture, and the PTY+MSSA co-culture (Figure 4), selected based on the differential biological activity exhibited by these co-cultures (Table 3). In the network, secondary metabolites found only in one extract are represented by circles, while metabolites identified in two extracts are indicated using square nodes, and triangular nodes signify that the metabolite was present in all three extracts. As seen in Figure 4, granatomycin D, granaticin, and dihydrogranaticin B are located in the same cluster on adjacent nodes due to their structural similarity. 

In addition to the more quantitative analysis of increased secondary metabolite production presented above, the molecular network also provides insight into possible explanations for the differential activity between the MRSA and MSSA co-cultures, namely that many naphthoquinone derivatives appear to be present only in the MRSA co-culture (five yellow circles). In addition, although the monoculture does produce some of these derivatives, there are many additional naphthoquinone metabolites produced in co-culture (yellow and blue circles, green squares) than in monoculture, thus indicating enhanced activation of this biosynthetic pathway when challenged with pathogen co-culture. Other distinct clusters and nodes are only found after *Streptomyces* sp. PTY087I2 was co-cultured with MRSA and MSSA. Further exploration may reveal that these previously silent biosynthetic gene clusters are being turned on to protect the bacterium from harmful pathogens.

## 3. Materials and Methods

### 3.1. Tunicate Collection and Bacterial Isolation

*Styela canopus*, a solitary tunicate, was collected from mangrove roots in Bastimentos Park, Bocas del Toro, Panama (9°17.398′ N 82°11.106′ W; Figure S1). The tunicate was surface sterilized with ethanol, washed with sterile seawater, homogenized, and plated onto a Reasoner’s 2A (R2A) agar (BD Difco™, Sparks, MD, USA) supplemented with 33 g/L Instant Ocean^®^ (Cincinnati, OH, USA) 80 mg/L cycloheximide, and 20 mg/L nalidixic acid to mimic sea water and reduce fungal contamination and fast growing bacteria. *Streptomyces* sp. PTY087I2 was then plated onto yeast starch peptone (YSP) agar (2/L g yeast extract, 5 g/L soluble starch, 1 g/L trypticase peptone, 33 g/L Instant Ocean^®^, and 8 g/L granulated agar), on which the bacterium exhibited morphological characteristics consistent with Actinobacteria as well as producing deep blue pigmentation (Figure S1).

### 3.2. Genomic Sequencing and antiSMASH Analysis of *Streptomyces* sp. PTY087I2

A draft genome for *Streptomyces* sp. PTY087I2 was obtained as reported elsewhere [27]. The draft genome assembly was deposited in DDBJ/EMBL/GenBank under accession number LZRD00000000. ORFcor v1.02 [33] and fasttree v2.1.7 [34] were used to compare with all other *Streptomyces* genomes held by the National Centre for Biotechnology Information (NCBI) (as of 22 February 2016). The genome was analyzed using antiSMASH 3.0.5 for identification of secondary metabolite clusters and percent similarity with known clusters [28]. 

### 3.3. *Streptomyces* sp. PTY087I2 Mono- and Co-Cultures

Cultures were prepared using previously described methods [29] with the following modifications. Prior to beginning, *Streptomyces* sp. PTY087I2 was grown on YSP agar, prepared as described above, for 3–4 days at 30 °C. On day zero, liquid seed culture of PTY087I2 was prepared by adding one PTY087I2 colony into 25 mL of YSP liquid media (prepared as described above without the addition of agar), and incubated for 72 h at 30 °C, 200 rpm. On day two, the four human pathogens [*Bacillus subtilis* (BS; ATCC 6051), methicillin-sensitive *Staphylococcus aureus* (MSSA; ATCC BAA-1718), methicillin-resistant *Staphylococcus aureus* (MRSA; ATCC BAA-1717), and *Pseudomonas aeruginosa* (PA; ATCC 15442)] were freshly streaked onto YSP agar. On day three, 1 mL of PTY087I2 seed culture (OD_600_ 4.0) was added to 25 mL of YSP liquid media in each of five separate 125 mL flasks, one for monoculture and one for each co-culture, and incubated for 24 h at 30 °C, 200 rpm. Concurrently on day three, one colony of each human pathogen was inoculated into 5 mL of YSP liquid media, and incubated for 24 h at 30 °C, 200 rpm. On day four, 200 μL of each human pathogen (OD_600_ 2.0) was added to the respective co-culture flask, and all flasks were incubated for six additional days at 30 °C, 200 rpm. On day ten, cultures were extracted as described below. Each culture was also streaked onto agar plates to confirm that co-cultures were dominated by *Streptomyces* sp. PTY087I2 as determined by the presence of aerial mycelia and production of deep blue pigmentation without observable contamination.

### 3.4. Extraction of *Streptomyces* sp. PTY087I2 Mono- and Co-Cultures

All extraction and HPLC grade solvents were purchased from Sigma Aldrich (St. Louis, MO, USA). Extracts were prepared as previously described [35]. Briefly, Diaion HP20 resin (Supelco) was pre-washed and cultures were sonicated to lyse cells. Resin was added to cultures (10% *w*/*v*) and allowed to incubate for 48 h at room temperature, shaking at 125 rpm. Cultures with resin were then filtered and washed with water to remove media components, after which they were extracted with methanol, dichloromethane, and acetone. Organic extracts were combined and dried in vacuo.

### 3.5. LC–MS and HRMS of *Streptomyces* sp. PTY087I2 Extracts

Bacterial extracts were prepared for liquid chromatography-mass spectrometry (LC–MS) at 1 mg/mL in HPLC grade methanol. An Agilent ESI single quadrupole mass spectrometer (Santa Clara, CA, USA) coupled to an Agilent 1260 high performance liquid chromatography (HPLC) system with a G1311 quaternary pump, G1322 degasser, and a G1315 diode array detector was used to obtain LC–MS data. A gradient elution was used from 25% acetonitrile with 0.1% formic acid (ACN_FA_) in H_2_O to 100% ACN_FA_ over 75 min, held at 100% ACN_FA_ for 5 min using an Eclipse XDB-C_18_ (4.6 × 150 mm, 5 μm) RP-HPLC column and a flow rate of 0.7 mL/min. Agilent Chemstation software vB04.02 was used to quantitate area under the curve (AUC) via manual integration of peaks at UV absorbance 254 nm.

High resolution mass spectrometry (HRMS) was obtained on a QStar Elite (Applied Biosystems/MDS SCIEX, Concord, ON, Canada) using an Inertsil ODS-3, 3 μM, 2.1 × 150 mm column and a flow rate of 0.12 mL/min. Samples were prepared at 1 mg/mL in HPLC grade methanol and run using gradient elution from 25% ACN_FA_/H_2_O to 100% ACN_FA_ over 55 min, held at 100% ACN_FA_ for 5 min.

### 3.6. Antibacterial Bioassays

*Streptomyces* sp. PTY087I2 monoculture and co-culture extracts were tested for antibacterial activity against the four human pathogens used in co-culture, including *Bacillus subtilis* (BS), methicillin-sensitive *Staphylococcus aureus* (MSSA), methicillin-resistant *Staphylococcus aureus* (MRSA), and *Pseudomonas aeruginosa* (PA). All bioassays were performed in 96-well flat bottom plates (Corning Costar™, Kennebunk, ME, USA) using Tryptic Soy Broth (BD Difco™, Sparks, MD, USA) for MSSA, MRSA, and PA, incubated at 37 °C, and using Nutrient Broth (BD Difco™, Sparks, MD, USA) for BA, incubated at 30 °C.

Bioassays were performed as previously described [35,36], with the following modifications: Pathogen inocula were adjusted to OD_600_ 0.1 (approximately 1–2 × 10^8^ CFU/mL, [37]) prior to use. A master mix was prepared using 1.6 mL adjusted bacterial inoculum, 7.84 mL sterile water, and 6.4 mL of broth. Master mix was aliquoted into each well (198 μL) to which was added 2 μL of either positive control [vancomycin (BA, MSSA, and MRSA) or gentamicin (PA), final testing concentration 2.5 μg/mL], negative control [dimethyl sulfoxide (DMSO)], or extract prepared in DMSO [screened at final concentration of 400 μg/mL; minimum inhibitory concentrations (MICs) performed using serial dilution]. Sterility was measured using wells containing 98 μL of sterile water, 100 μL of broth, and 2 μL of DMSO. Samples and controls were tested in technical triplicates with at least two experimental repeats. Plates were read at 0 and 24 h at 600 nm using a Synergy Hybrid Reader (Biotek, Winooski, VT, USA). Results were compared to the DMSO negative control.

### 3.7. Molecular Networking of *Streptomyces* sp. PTY087I2 Extracts

Extracts of the *Streptomyces* sp. PTY087I2 monoculture, PTY087I2 co-cultured with MRSA, and PTY087I2 co-cultured with MSSA were analyzed via LC–MS/MS on a QStar Elite (Applied Biosystems/MDS SCIEX, Concord, ON, Canada) coupled to a Shimadzu LC-10ADvp HPLC pump and an SIL-HTa autosampler using an Inertsil ODS-3, 3 μM, 2.1 × 150 mm column at a flow rate of 0.7 mL/min. Samples were prepared at 1 mg/mL in HPLC grade methanol and run using gradient elution from 40% ACN_FA_/H_2_O to 100% ACN_FA_ over 30 min. Parameters for electrospray ionization (ESI) were source voltage HV, 5500 V; nebulizing gas source 1 (GS1), 25 psi; GS2, 15 psi; capillary temperature 200 °C; declustering potential (DP), 60 V. Data were obtained using an information dependent acquisition (IDA) method with three scan events including a positive MS survey scan, ranging from *m/z* 300–2000, then two data-dependent MS/MS scans of the first and second most intense ions from the first scan event. MS/MS settings included dynamic background subtract, automatic collision energy, and automatic MS/MS accumulation with fragment intensity multiplier 2.0 and max accumulation of 2.0 s.

Molecular networks were generated as previously described [32,38], with the following modifications: Data were converted to mzXML format using ProteoWizard package [39] and subsequently analyzed in GnPS: Global Natural Products Social Molecular Networking (http://gnps.ucsd.edu). A minimum cosine similarity score of 0.65 with at least four matching peaks, parent mass tolerance of 1.0 Da, and fragment ion tolerance 0.3 Da were selected to generate consensus spectra. Files were imported into Cytoscape 2.8.3 and nodes were arranged with FM3 layout plugin [40].

## 4. Conclusions

With the relative ease and reduced cost now associated with bacterial genome sequencing, it is increasingly obvious that there are vast numbers of secondary metabolite biosynthetic gene clusters present in marine bacteria that are not expressed in laboratory settings. New strategies, such as mixed fermentation, are needed to induce production of these silent biosynthetic pathways, even in previously well-studied genera such as *Streptomyces*. Co-cultures with small amounts of challenge pathogens may provide environmental stressors that promote increased biosynthesis of active antibacterial compounds. The tunicate-associated bacterium, *Streptomyces* sp. PTY087I2, exhibited enhanced production of secondary metabolites and increased antibacterial activity when co-cultured with the human pathogens *Bacillus subtilis*, methicillin-sensitive *Staphylococcus aureus* (MSSA), methicillin-resistant *Staphylococcus aureus* (MRSA), and *Pseudomonas aeruginosa*. The enhanced biological activity was determined to be the result of increased production of three naphthoquinone derivatives, granatomycin D, granaticin, and dihydrogranaticin B, particularly when PTY087I2 was co-cultured with MRSA. Thus, co-culturing with human pathogens may be one strategy for inducing antibiotic secondary metabolite production from previously silent biosynthetic pathways under laboratory conditions. Further mixed fermentation experiments should explore: the effect of varying the co-culture ratios; optimum timing of pathogen inoculation; and methods to increase production of compounds with activity against Gram-negative pathogens.

## Figures and Tables

**Table 1:** Biosynthetic gene clusters identified in the *Streptomyces* sp. PTY087I2 genome using antibiotics and Secondary Metabolite Analysis SHell (antiSMASH) [28].

Cluster	Type	Most Similar Known Cluster	Percent Similarity	Molecular Weight (g/mol) ^a^
1	Bacteriocin	Tetronasin	3%	602.80
2	Nrps	Coelibactin	72%	391.46
3	Nrps-T1pks	SGR PTMs	100%	
4	Otherks-Nrps	Calcium-dependent antibiotic	15%	
5	Terpene	Hopene	69%	410.72
6	Bacteriocin	-	-	
7	Terpene	-	-	
8	Lantipeptide	-	-	
9	Melanin	Melanin	100%	318.28
10	T3pks	Herboxidiene	8%	438.60
11	Nrps	Herboxidiene	9%	438.60
12	Nrps-T1pks	Daptomycin	4%	1620.67
13	Ectoine	Ectoine	100%	142.16
14	Siderophore	Kinamycin	22%	484.50
15	T2pks	Granaticin	83%	444.39
16	Butyrolactone	Oxazolomycin	6%	655.78
17	Terpene	-	-	
18	Nrps	Griseobactin	100%	1179.50
19	Otherks-T1pks	Nataxazole	59%	400.38
20	T3pks	Herboxidiene	6%	438.60
21	Nrps	Coelichelin	72%	565.58
22	Lantipeptide	Guadinomine	7%	518.57
23	Thiopeptide-Lantipeptide	-	-	
24	Lassopeptide	-	-	
25	Nrps	Phosphonoglycans	3%	
26	Lassopeptide	SRO15-2005	100%	
27	Nrps	SRO15-2005	100%	
28	Terpene	Steffimycin	19%	574.53
29	Siderophore	Desferrioxamine B	80%	560.68
30	Ectoine	Pristinamycin	23%	IA 866.96; IB 852.93; IIA 525.59; IIB 527.62
31	Lantipeptide-Melanin	Labyrinthopeptin A1, A2, A3	40%	A1 2073.76; A2 1922.68; A3 2188.78
32	T1pks-Nrps	Enduracidin	10%	2355.61
33	Ladderane-Arylpolyene	Skyllamycin	26%	1481.99
34	Other	Skyllamycin	26%	1481.99
35	Lantipeptide	AmfS	100%	
36	Nrps	Skyllamycin	10%	1481.99
37	Nrps	-	-	

^a^ Molecular weight provided for first compound in a series unless derivative specified by antiSMASH.

**Table 2:** Area under curve (AUC) of Liquid Chromatography–Mass Spectrometry (LC–MS) peaks, A–G, from *Streptomyces* sp. PTY087I2 monoculture and co-culture with human pathogens (see Figure S2 for chromatograms; BS = *Bacillus subtilis*; MSSA = methicillin-sensitive *Staphylococcus aureus*; MRSA = methicillin-resistant *Staphylococcus aureus*; PA = *Pseudomonas aeruginosa*).

Extract	Peak A (5 min)	Peak B (10 min)	Peak C (11 min)	Peak D (13 min)	Peak E (16 min)	Peak F (34 min)	Peak G (68 min)
Monoculture	930.4	231.3	15.1	13.2	5.4	27.3	29.0
Co-culture (BS)	2117.8	826.0	208.4	149.7	58.7	86.6	110.7
Co-culture (MSSA)	1480.5	1107.4	83.6	25.9	38.2	84.2	81.9
Co-culture (MRSA)	962.2	463.2	349.3	206.2	139.6	56.4	67.1
Co-culture (PA)	739.0	335.1	87.4	49.7	39.3	33.3	33.8

**Table 3:** MIC values (μg/mL) for *Streptomyces* sp. PTY087I2 monoculture and co-culture extracts against human pathogens used in co-cultures (BS = *Bacillus subtilis*; MSSA = methicillin-sensitive *Staphylococcus* *aureus*; MRSA = methicillin-resistant *Staphylococcus aureus*; PA = *Pseudomonas aeruginosa*).

Extract	*Bacillus subtilis*	MSSA	MRSA	*Pseudomonas aeruginosa*
Monoculture	25	50	50	>400
Co-culture (BS)	6.25	25	25	>400
Co-culture (MSSA)	25	50	50	>400
Co-culture (MRSA)	1.56	12.5	6.25	>400
Co-culture (PA)	6.25	12.5	12.5	>400

## Supplementary Materials

The following are available online at www.mdpi.com/1660-3397/15/8/250/s1: Photos of *Styela canopus*, *Streptomyces* sp. PTY087I2, LC–MS overlays of mono- and co-cultures, HRMS of peaks C and D, and granaticin biosynthetic gene comparison.
