# Supplementary material for: Upregulation and Identification of Antibiotic Activity of a Marine-Derived Streptomyces sp. via Co-Cultures with Human Pathogens
Source: Mar Drugs. 2017 Aug 11;15(8):250. doi: 10.3390/md15080250 (PMC5577605; doi:10.3390/md15080250)
Supplement: Supplementary file 1 [file marinedrugs-15-00250-s001.pdf]

Supporting Information

Upregulation and Identification of Antibiotic Activity of a Marine-Derived *Streptomyces* sp. via Co-Cultures with Challenge Pathogens

Anne A. Sung, Samantha M. Gromek and Marcy J. Balunas*

Division of Medicinal Chemistry, Department of Pharmaceutical Sciences, University of Connecticut, Storrs, Connecticut 06269, USA

List of Supporting Information:

Figure S1. *Styela canopus*, the solitary tunicate collected from mangrove roots in Bastimentos Park, Bocas del Toro, Panama and *Streptomyces* sp. PTY087I2 a bacterium isolated from this tunicate, on YSP+IO agar plate and in YSP+IO liquid media

Figure S2. UV chromatogram overlays of monoculture with each co-culture demonstrating upregulation in secondary metabolite production

Figure S3. High resolution mass spectral confirmation of granatomycin D, granaticin, and dihydrogranaticin B production by *Streptomyces* sp. PTY087I2

Table S1. Granaticin biosynthetic genes from *Streptomyces* sp. PTY087I2 and the % identity to those from *S. violaceoruber* Tu22

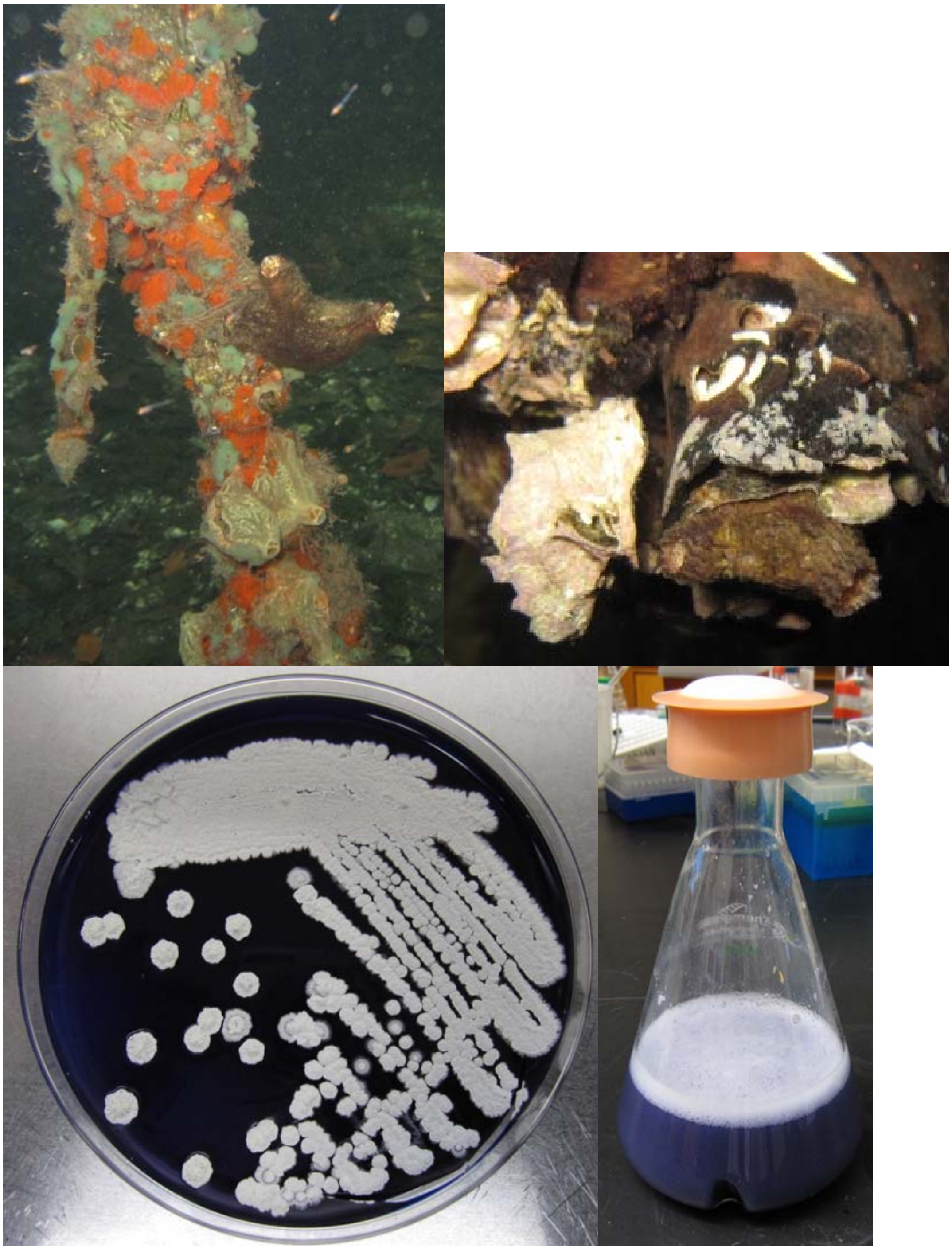

Figure S1. ***Styela canopus*****, the solitary tunicate collected from mangrove roots in Bastimentos Park, Bocas del Toro, Panama (top) and** ***Streptomyces*** **sp. PTY087I2 a bacterium isolated from this tunicate, on YSP+IO agar plate (left bottom) and in YSP+IO liquid media (right bottom).**

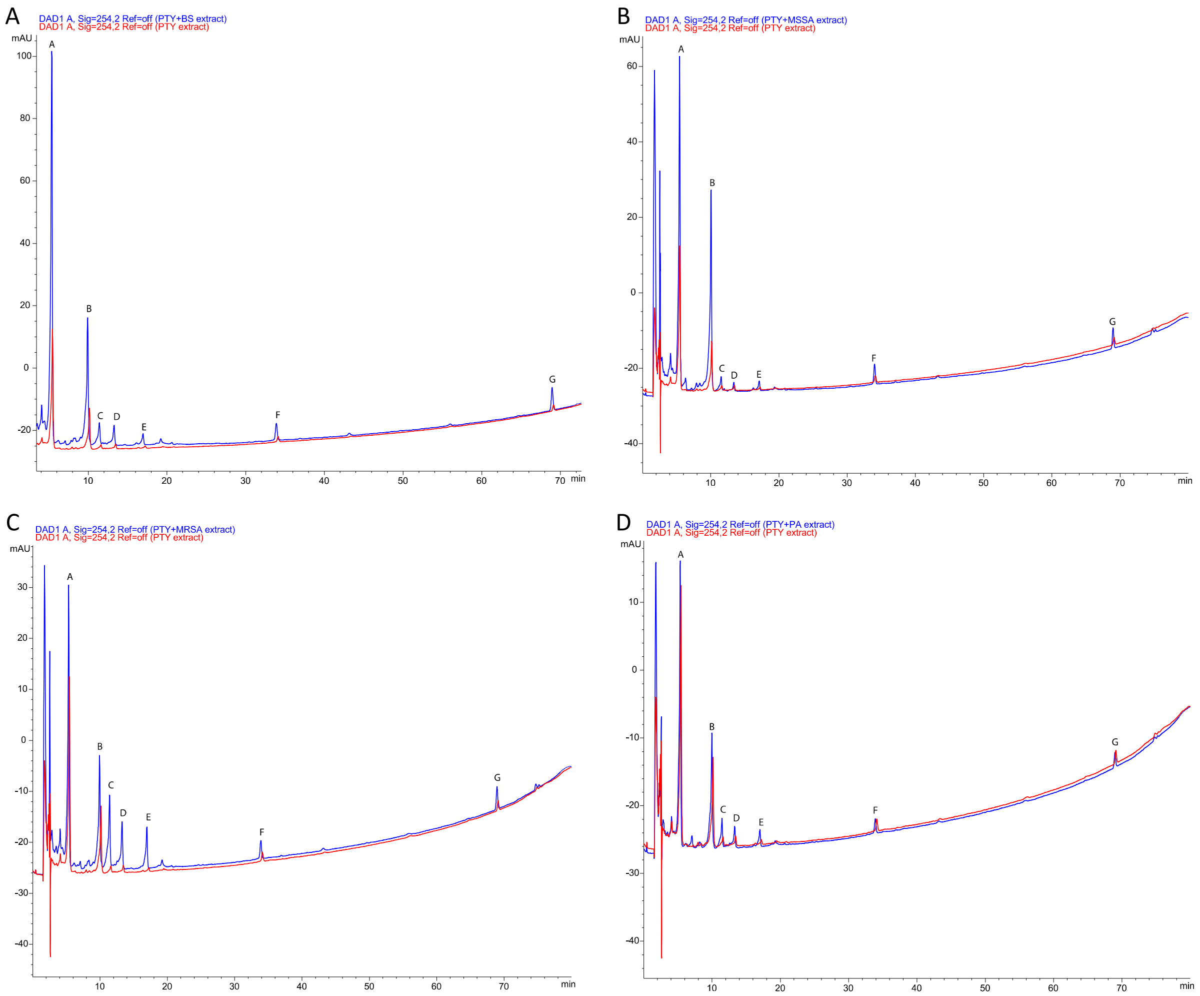

Figure S2. UV chromatogram overlays of monoculture with each co-culture demonstrating upregulation in secondary metabolite production. (A) Monoculture (red) and co-culture with *Bacillus subtilis* (BS, blue); (B) monoculture (red) and co-culture with methicillin sensitive *Staphylococcus aureus* (MSSA, blue); (C) monoculture (red) and co-culture with methicillin resistant *Staphylococcus aureus* (MRSA, blue); and (D) monoculture (red) and co-culture with *Pseudomonas aeruginosa* (PA, blue).

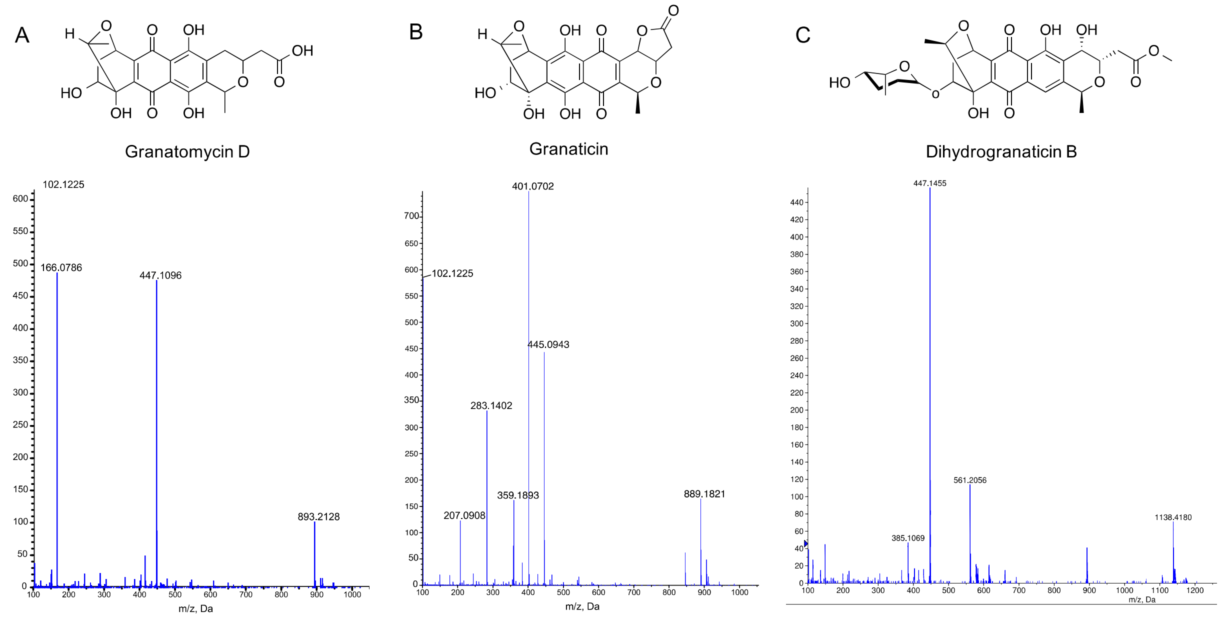

Figure S3. High resolution mass spectral confirmation of granatomycin D, granaticin, and dihydrogranaticin B production by *Streptomyces* sp. PTY087I2. High resolution mass spectral data was collected on Qstar Elite system as described in the Materials and Methods. (A) Granatomycin D (stereochemistry not defined in literature) eluted at retention time (t_R) 11.4 min. The *m/z* of $[M+H]^+$ 447.1096 is consistent with a molecular formula of $C_{22}H_{22}O_{10}$, confirming presence of granatomycin D. **(**B) Granaticin eluted at t_R 13.2 min. The *m/z* of $[M+H]^+$ 445.0943 is consistent with a molecular formula of $C_{22}H_{20}O_{10}$, confirming presence of granaticin. (C) Dihydrogranaticin B eluted at retention time (t_R) 17.0 min. The *m/z* of $[M+H]^+$ 561.2056 is consistent with a molecular formula of $C_{28}H_{32}O_{12}$, confirming presence of dihydrogranaticin B.

Table S1. Granaticin biosynthetic genes from *Streptomyces* sp. PTY087I2 and their % identity to those from *S. violaceoruber* TÜ22 (ORF, gene name, and deduced role from [1]).

ORF	Gene	Deduced role	% identity
7	*pkaA*	Serine threonine protein kinase	72
8	-	-	n.p.
9	*actII*-4	Pathway-specific transcriptional activator	n.p.
10	*degU*	Response regulator oft wo-component system	n.p.
11	*degS*	Sensor kinase of two-component system	n.p.
12	-	-	n.p.
13	-	-	n.p.
14	*dnrS*	Glycosyl transferase	93
15	*actII*-2	Transmembrane protein	84
16	*strD*	dTDP-1-glucose synthase	84
17	*strE*	dTDP-glucose-4,6-dehydratase	84
18	*actVI*-3	Cyclase dehydratase	77
19	-	Disulphide bond forming protein	76
20	*soxR*	Transcriptor activator in redox cotrol	n.p.
21	*actVA*-5	Hydroxylase	79
22	*dnmV*	dTDP4-keto-6-deoxyhexose-reductase	80
23	*rfbH (ascC)*	CDP-4-keto-6-deoxyglucose-3-dehydratase E1	94
24	-	-	n.p.
25	*strM*	dTDP-4-keto-6-deoxyglucose-3,5-epimerase in streptomycin biosynthesis	87
26	*rdmF*	Rhodomycin biosynthesis	n.p.
27	*dnmT*	dTDP-4-keto-6-deoxyglucose-2,3-dehydratase	91
28	*actVA*-3	unknown	n.p.
29	*lmbY*	FMN dependent monooxygenase in lincomycin biosynthesis	91
6	*actIII*	Keto reductase	94
5	*actIII*	Keto reductase for C-9	
1	*actI*-1	Keto acyl synthase	95
2	*actI*-2	Chain Length Factor	80
3	*actI*-3	Acyl carrier protein	80
4	*actVII*	First ring aromatisation (aromatase)	85
30	*actVA*-3	Unknown	n.p.
31	*actVI*-A	Unknown	n.p.
32	-	-	n.p.
33	*actIV*	Second ring cyclisation (cyclase)	83
34	*actVB*	FMN:NADH oxioreductase	82
35	Unknown	-	n.p.
36	Unknown	-	n.p.
37	*nshA*	Transcriptional activator	n.p.
	-	tRNA-ala	92
	-	YcaO-like family protein	77

n.p. = not present in *Streptomyces* sp. PTY087I2; 1. Ichinose, K.; Bedford, D.J.; Tornus, D.; Bechthold, A.; Bibb, M.J.; Revill, W.P.; Floss, H.G.; Hopwood, D.A. The granaticin biosynthetic gene cluster of *streptomyces violaceoruber* tu22: Sequence analysis and expression in a heterologous host. *Chem. Biol.* **1998**, *5*, 647-659.
